# Study on the Isolated Asphaltene Thermal Cracking from an Unconventional Oil Using Diverse Estimating Arrhenius Parameter Approaches

**DOI:** 10.3390/molecules30224468

**Published:** 2025-11-19

**Authors:** Guillermo Félix, Alexis Tirado, Mikhail A. Varfolomeev, Eder Lugo-Medina, Carlos A. Soto-Robles, Jorge Ancheyta

**Affiliations:** 1 Department of Petroleum Engineering, Kazan Federal University, Kazan 420008, Russia; atirado.kota@kpfu.ru (A.T.); vma.ksu@gmail.com (M.A.V.); 2 IT de Los Mochis, Tecnológico Nacional de México, Los Mochis 81259, Mexico; eder.lm@mochis.tecnm.mx; 3 Centro Universitario de Los Altos, Universidad de Guadalajara, Av. Rafael Casillas Aceves No. 1200, Tepatitlán de Morelos 47620, Mexico; carlos.soto@academicos.udg.mx; 4 Unidad de Posgrado y Alta Investigación en Materiales, Escuela Superior de Ingeniería Química e Industrias Extractivas, Instituto Politécnico Nacional, Mexico City 07738, Mexico

**Keywords:** kinetic model, heavy crude oil, pyrolysis, asphaltenes

## Abstract

The lack of complex schemes and proper kinetic studies for asphaltenes pyrolysis is a significant problem for understanding the reaction mechanisms. Therefore, this study evaluates various parameter estimation approaches (focusing on reaction rate coefficients and Arrhenius parameters), objective functions (sum of squared errors and average absolute errors), and reaction schemes (4-lump and 6-lump) for kinetic modeling of asphaltene pyrolysis. The reaction schemes include asphaltenes, maltenes, gases, and coke, as well as the fractionation of maltenes into polar aromatics, naphthene aromatics, and saturates. Both networks showed adequate accuracy, but the 6-lump model improves yield predictions. Using Method 1 (optimizing reaction rate coefficients) and the sum of square errors as the objective function yields the best accuracy. The analyses confirm that temperature influences selectivity: lower temperatures (below 390 °C) favor the generation of maltenes and gases, while higher temperatures (above 390 °C) promote coke formation. Extended high-temperature exposure leads to secondary cracking of maltenes. Furthermore, the key transformations include the cleavage of aliphatic chains, the aromatization of saturated aromatics, and the polyaddition of free radicals.

## 1. Introduction

Pyrolysis is a widely adopted technology for upgrading heavy oil, primarily due to its economic advantages and manageable operational complexities. This method is particularly advantageous because it requires relatively lower capital investment than other upgrading techniques. Additionally, the operational issues involved in pyrolysis are less intricate, allowing for simple implementation and maintenance [1,2,3]. However, the presence of high-molecular-weight fractions, particularly asphaltenes, presents an essential challenge in converting heavy feedstocks. The high content of polyaromatic cores in these compounds leads to solidification and increased viscosity, which complicates the recovery and processing of heavy and extra-heavy crude oils. The reason is that asphaltenes contain a larger proportion of heteroatoms, such as sulfur, nitrogen, and oxygen, which play a crucial role in their reactivity [4,5,6,7].

Understanding the behavior and chemistry of the asphaltene fraction is equally vital, as they can influence the overall performance and efficiency of upgrading processes. Addressing the reaction mechanism of high-molecular-weight components is crucial for improving the quality of heavy petroleum products [8,9,10,11]. Performing kinetic studies related to the upgrading of heavy crude oil and its fractions is a suitable option for understanding the reaction mechanisms. However, the complexity arises from the extensive diversity of chemical compounds within heavy petroleum, which undergo several reactions. As a result, researchers frequently implement the lumping approach, which integrates similar chemical compounds into broader groups known as pseudocomponents. This method facilitates predicting the distribution and behavior of these chemical species during upgrading processes [12,13,14,15].

A critical factor in the accuracy of the lumping approach lies in the selection and appropriate representation of the reaction scheme involving all the pseudocomponents. This is typically accomplished by considering all plausible reaction pathways between contemplated hydrocarbons, the nature of all hydrocarbons, and their chemical transformations. Furthermore, a proper methodology for parameter estimation is another key aspect of the underlying realistic mechanisms under specific operational conditions [16,17,18,19]. Pyrolysis significantly reduces the boiling point and molecular weight of feedstock, involving complex chemical mechanisms. These key mechanisms mainly include the free radical and electron transfer mechanisms. These thermally driven reactions lack selectivity in bond cleavage, with the weakest bonds breaking first. The fundamental chemical transformations during pyrolysis include homolytic cleavage of C–C bonds, side-chain fragmentation, ring growth, hydrogen shuttling, hydrogenation of aromatics, dehydrogenation of cycloparaffins, and the removal of heteroatoms and metals. Among these, the homolytic cleavage of C–C bonds is a crucial reaction, characterized by a free radical mechanism [1,20,21,22].

Although various kinetic models based on the lumping approach have been proposed for heavy oil upgrading under pyrolysis conditions [23,24,25,26,27,28,29,30,31,32,33,34,35,36,37,38], only a few are focused on the kinetics of asphaltene pyrolysis individually [30,32,33]. However, in these models, the uncomplicated reaction networks deal with a low number of compounds, lacking significant pathways that represent the chemical phenomena of asphaltenes. Therefore, developing an exhaustive and precise kinetic model and contemplating the possible pathways that asphaltenes undergo under these conditions is indispensable. In this work, different reaction schemes (4- and 6-lump) based on SARA fractions are evaluated for the pyrolysis of isolated asphaltenes from heavy crude oil. Diverse approaches for estimating kinetic parameters are examined by optimizing the reaction rate coefficients and Arrhenius parameters.

## 2. Results and Discussion

### 2.1. Evaluation of the Optimization Approach

**Figure 1:**
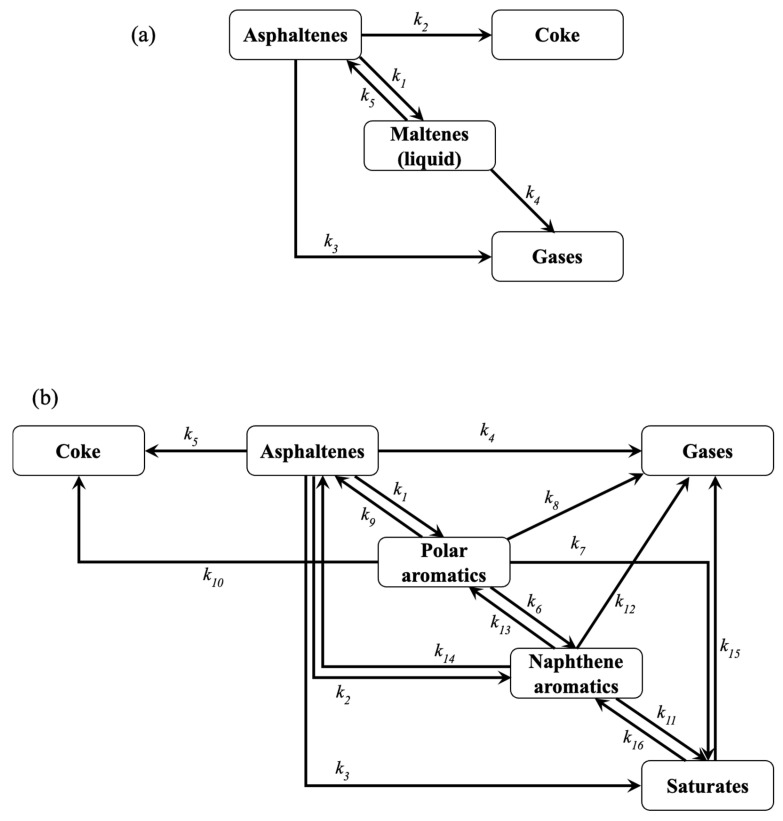
4-lump (**a**) and 6-lump (**b**) reaction schemes for asphaltene pyrolysis.

The parameter estimation methodology is applied to all different approaches (Methods 1, 2, and 3) using two different OF for the 4-lump kinetic model (Figure 1a). Table 1 reports the estimated reaction rate coefficients with three methods using AAE as OFs for this network. The kinetic parameters for asphaltene conversion exhibit higher values than the transformation of maltenes, which is an expected behavior due to the larger number and susceptibility of asphaltene molecules, rather than maltenes, together with the higher yield of the former. The results of the kinetic parameters imply that the transformation of asphaltenes is toward maltenes and gas at low temperatures (330–360 °C), according to larger values for the corresponding reactions. This coincides with Savage et al. [39], where the maltene and gas fractions were the principal products of asphaltene pyrolysis. However, when the temperature surpasses 420 °C, the reaction rate coefficients to produce coke and gas from asphaltenes remarkably increase, these being the predominant reactions. The peri-condensation transformations, where the number of quaternary carbons in aromatic cores remains almost unchanged but the attached aliphatic chains are reduced, require large activation energy; thus, these reactions are favored at high temperatures [40,41,42]. Based on the reaction rate coefficients obtained for Method 1, maltenes are preferably over-cracked rather than recombined to form asphaltenes, which agrees with other results [39,43], where the secondary cracking of maltenes generates lighter liquid molecules and gases. The calculated activation energies displayed larger values for asphaltene decomposition than those of maltenes, specially producing coke from the former. Alhumaidan et al. [44] reported that as the temperature of pyrolysis increases, the asphaltene molecules become more refractory, increasing the necessary energy to decompose. The lowest activation energy corresponds to the gas production due to maltene disintegration.

The optimized reaction rate coefficients using Method 2 suggest that asphaltenes are mainly transformed into maltenes and gas under temperatures of 420 °C. These results are like Method 1 but with a wider range of temperatures, mainly producing maltenes and gas from asphaltenes. Nonetheless, as the temperature reaches 450 °C, the largest kinetic parameter is for coke formation from asphaltene agglomeration since this latter reaction is enhanced as the temperature is raised. Regarding maltene conversion, the reaction rate coefficients imply that these compounds are only recombined into asphaltenes, and there is no gas production from maltenes. This is an unexpected outcome because the over-cracking of secondary maltenes to form gas is usually performed at asphaltene pyrolysis conditions [32,39,45]. The calculated activation energies for Method 2 are similar to those for Method 1, with a larger value of the activation energy corresponding to the reaction of maltenes to gas. The reason is that the optimized kinetic parameters in this approach indicate that more energy is needed to perform the secondary cracking of generated maltene molecules, which does not agree with other results.

The reaction rate coefficients determined using Method 3 indicate that the production of asphaltenes to maltenes and gases, and in turn maltenes to gases, prevail at temperatures below 400 °C. This is attributed to the thermally susceptible heteroatomic compounds within asphaltenes undergoing cracking reactions. Additionally, these latter and secondary lower molecular weight hydrocarbons (maltenes) suffer dealkylation reactions [39,46,47,48]. However, when the temperature surpasses 400 °C, the coke formation through asphaltene polyaddition is enhanced. Various works [39,45,49] have reported the same trend, where the asphaltene transformation rapidly increases after these conditions, producing a large content of coke. The estimated activation energies for Method 3 display similar trends to those for Method 1. Among the three approaches, Method 1 exhibits the lowest values of AAE, indicating that this optimization technique accurately estimates the kinetic parameters to a higher degree than the others.

The optimized kinetic parameters for the 4-lump reaction scheme using Methods 1, 2, and 3 and SSE as OF are presented in Table 2.

The values obtained for the kinetic parameters using Method 1 imply that the conversion of asphaltenes is toward gas production at 330 °C. Boytsova et al. [50] reported that around 320 °C, the main transformation of asphaltenes is due to the dealkylation of aliphatic hydrocarbons located in exterior sites of the asphaltene structure. Nonetheless, at temperatures over 390 °C, the transformation of asphaltenes into maltenes and gases predominates, associated with the increase in thermal cracking rate. A similar trend is observed for maltene conversion since at 330 and 360 °C gases are principally produced from this fraction, but exceeding a temperature of 390 °C, the recombination of maltenes into asphaltenes prevails. This coincides with the same agglomeration reactions of free radicals produced by asphaltenes, indicating an in-series polyaddition route from maltenes to asphaltenes to coke, as proposed by Yasar et al. [32]. Regarding the calculated activation energies using this approach, larger values are obtained for recombination reactions (asphaltenes to coke, and maltenes to asphaltenes), which is an expected behavior due to the required energy to increase the thermal cracking rate. Meanwhile, the maltene and gas production from asphaltenes exhibit lower activation energies. The reason is that these reactions do not demand high energy to be performed and prevail at low temperatures.

For Method 2, the estimated kinetic parameters denote a higher rate of coke formation from asphaltenes at low temperatures (330 and 360 °C), but as the temperature is increased, the maltene production is enhanced. This is not an expected behavior since coke formation from asphaltene pyrolysis is low at temperatures under 400 °C. The values of these reaction rate coefficients also suggest that the thermal cracking of maltenes only produces asphaltenes by polyaddition reactions since the gas production from this fraction is not carried out. Usually, at pyrolysis conditions, the contrary trend is observed because the dealkylation of aromatic hydrocarbons to generate gas predominates, especially in archipelago-type asphaltenes [45,47,50]. Larger values of the calculated activation energies are shown in this approach for the reactions between asphaltenes and maltenes (forward and backward). This is uncommon for the forward reaction since although the generation of maltenes from asphaltenes is promoted as the temperature increases, it prevails at low and mild conditions [32]. The optimized kinetic parameters using Method 3 showed larger values for converting asphaltenes to coke and maltenes. Meanwhile, this latter fraction is only converted into gases since the recombination of these molecules into asphaltenes is barely carried out according to the estimated reaction rate coefficients. Akmaz et al. [45] reported similar outcomes for asphaltene pyrolysis, where prolonging the reaction time and increasing the temperature causes over-cracking of asphaltenes and secondary thermal decomposition of maltenes to generate coke and gas. Similar to the last approach using this method, larger activation energies are obtained for the reactions of asphaltenes and maltenes, displaying the lowest value for the gas generation (from asphaltenes and maltenes). Also, when AAE is used as OF, applying SSE and Method 1 exhibits better accuracy in predicting the product yields. The reason may be attributed to the larger number of parameters optimized at each temperature in Method 1 compared with those estimated for all temperatures in Methods 2 and 3. This allows for a more accurate representation of reactions performed at different temperatures since optimizing activation energies describes an average behavior of all performed reactions.

A critical finding of our analysis is that the choice of the OF exerts a profound influence on the statistical reliability of the estimated kinetic parameters, as evidenced by their confidence intervals (CIs) in Table 1 and Table 2. When the AAE was employed as the OF, the resulting CIs were excessively wide (Table 1), indicating a severe degree of multicollinearity among the parameters. Under such conditions, these intervals are statistically non-representative and offer little practical precision. This phenomenon is mechanistically explained by the role of the SSE. The AAE objective function does not minimize the SSE, which is a fundamental component in the calculation of the standard errors. Consequently, the unmitigated SSE propagates through the error analysis, raising the standard errors and, in turn, yielding the vast, uninformative CIs. In direct contrast, using the SSE itself as the objective function actively minimizes this source of uncertainty, leading to a substantial reduction in the standard error and producing the tight, statistically representative CIs observed in Table 2.

All the estimated kinetic parameters using each approach agree that the production of lighter compounds prevails at temperatures lower than 390 °C. After this, the recombination reactions predominate for asphaltenes and maltenes, generating a large amount of coke as an end-product. This performance is attributed to the free radical formation through thermal cracking from aromatic cores within asphaltenes and maltenes [51,52,53]. However, this can also be attributed to the dissolving medium because when these heavy fractions (asphaltenes) are dissolved within the colloidal heavy oil mixture, they are surrounded by partial polar compounds (resins), which enhance their interactions with non-polar compounds [54,55,56,57]. Nonetheless, in this work, the pyrolysis of individual asphaltenes is performed without any dissolvent compound that improves the interaction between polar and non-polar compounds. These phenomena can cause polar fractions to agglomerate each other, provoking the growth of highly condensed aromatic cores, such as coke. This mechanism is performed through the overlaying of several asphaltene molecules exchanging different charges, such as π-π bonding [58,59].

**Figure 2:**
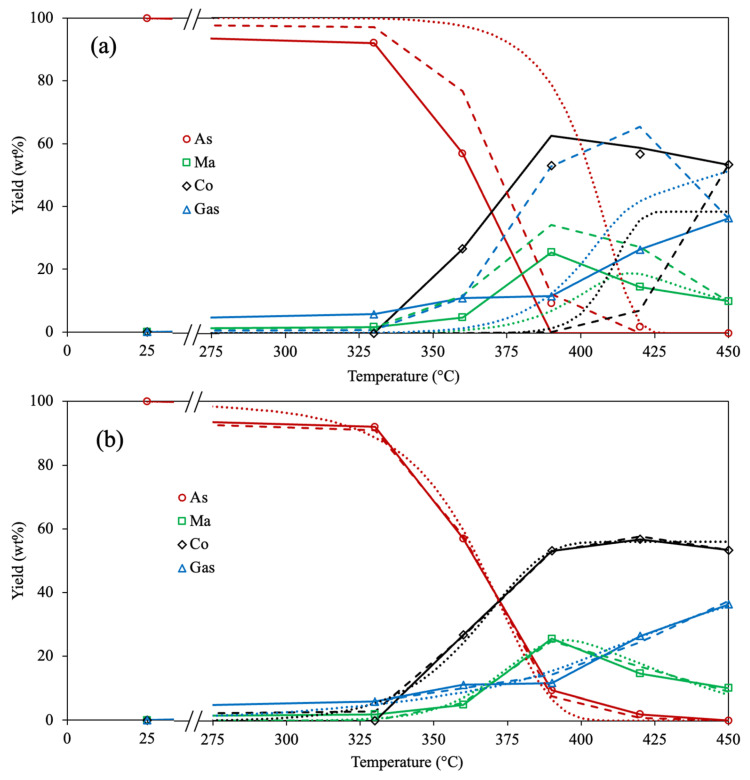
Experimental (symbols) and calculated (lines) yields for all involved compounds in the 4-lump reaction scheme with Method 1 (solid), Method 2 (dashed), and Method 3 (dotted) using (**a**) AAE and (**b**) SSE as the OF.

Figure 2 depicts the experimental and calculated yields of involved compounds for the 4-lump reaction network using all optimizing approaches (Methods 1, 2, and 3) and objective functions (AAE and SSE). The analysis of the experimental data demonstrates that asphaltene conversion is increased with temperature, being almost depleted at 450 °C. Similar results are reported by Savage et al. [39] since an entire asphaltene pyrolysis conversion is achieved at 450 °C and 30 min. The yield of maltenes rises with temperature until 390 °C, then the amount of this compound is reduced. Akmaz et al. [45] observed that increasing the temperature from 425 °C to 450 °C diminishes the amount of maltenes produced after asphaltene pyrolysis. Likewise, Yasar et al. [32] found that the yield of maltenes is reduced after surpassing a temperature of 425 °C. These outcomes suggest that the rate of secondary pyrolysis of generated maltenes increases after 400 °C, causing recombination reactions on these molecules to form asphaltenes and subsequently coke. The gas generation is linearly incremented with the upsurge of temperature, attributed to the scission of peripheral hydrocarbon chains within asphaltenes at the primary stage and the secondary thermal cracking of products at the prolonged stage [39,45,60]. The yield of coke is negligible at 330 °C, which agrees with the results reported by Savage et al. [39], where no coke was obtained in the asphaltene pyrolysis at 350 °C. The coke formation is exponentially raised as the temperature increases from 360 °C up to 420 °C; after this, the yield of coke remains almost constant, which agrees with the low amount of asphaltenes under these conditions. The cause may be due to dealkylation, dehydrogenation, and aromatization reactions, provoking a hydrogen deficit in asphaltene structure as the temperature rises, producing highly polar free radicals and subsequently coke [40,45,61]. However, the concentration of condensed aromatic cores within the asphaltene fraction diminishes progressively with decreasing yield, leveling off the coke yield over 390 °C. Based on these outcomes and the estimated kinetic parameters, the asphaltene pyrolysis comprises diverse reactions: the scission of peripheral aliphatic chains on aromatic rings, the dehydrogenation of saturated cores, the recombination of free radicals, and the peri-condensation of highly aromatic cores.

In Figure 2a, the predicted yields of all fractions employing the diverse optimization approaches (Methods 1, 2, and 3) and the AAE as OF are shown. The lowest difference between experimental and calculated data is obtained using Method 1, especially for the coke fraction. This can be attributed to the difficulty of representing the formation of this latter compound using the Arrhenius parameters because it demands a high quantity of energy to be performed. Furthermore, the rate of this reaction grows exponentially and not linearly with temperature, as the reaction rate coefficients need to increase in the Arrhenius equation [62,63]. The predicted yields using Methods 1, 2, and 3, together with the SSE as OF, are depicted in Figure 2b. Similar outcomes are obtained because Method 1 exhibits better accuracy than Methods 2 and 3. However, the SSE as OF displays a lower fitting difference among all optimization approaches. The reason may be associated with the fact that a small change in the kinetic parameter causes a big percentage error using AAE as OF, compared with the small difference in yields using SSE as OF. Therefore, by comparing all the results, using the SSE as OF showed enhanced precision for calculating the corresponding yield for all optimization methods, with Method 1 being the most accurate.

**Figure 3:**
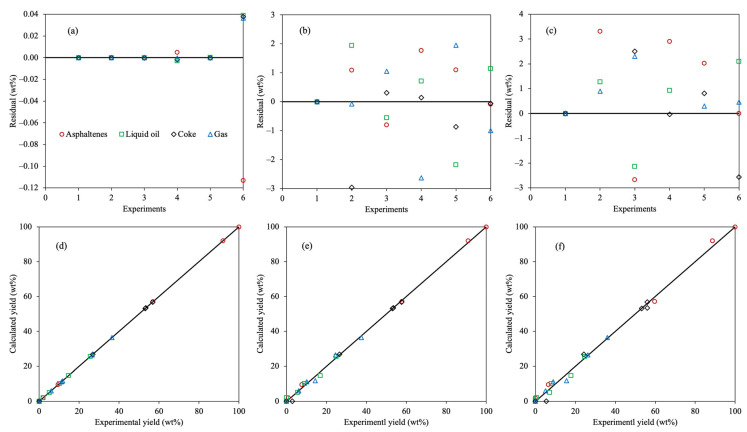
Residual (**a**–**c**) and parity (**d**–**f**) plots for the calculated 4-lump kinetic models using Method 1 (**a**,**d**), Method 2 (**b**,**e**), and Method 3 (**c**,**f**) with SSE as the OF.

The statistical analysis for the estimated kinetic model using the 4-lump reaction scheme, all optimization methods, and the SSE objective function are presented in Figure 3.

The residual plots using all approaches did not show any trend to over- or underestimate the experimental yields, indicating that the parameter estimation methodology using all methods is appropriate. Among all residuals, those applying Method 1 exhibit the lowest values, which coincides with better accuracy using this approach. Methods 1 and 3 agree that the furthest residuals are estimated for the asphaltenes yields, concerning the larger yield of this fraction than the others. However, maltene and gas fractions showed a greater discrepancy between the experimental and calculated data using Method 2, which may be due to the uncommon trends suggested by the corresponding kinetic parameter obtained using this approach. The parity plot agrees with the results obtained in Table 2, where Method 1 displays an enhanced accuracy because of the linear correlation obtained between the calculated and experimental data.

**Figure 4:**
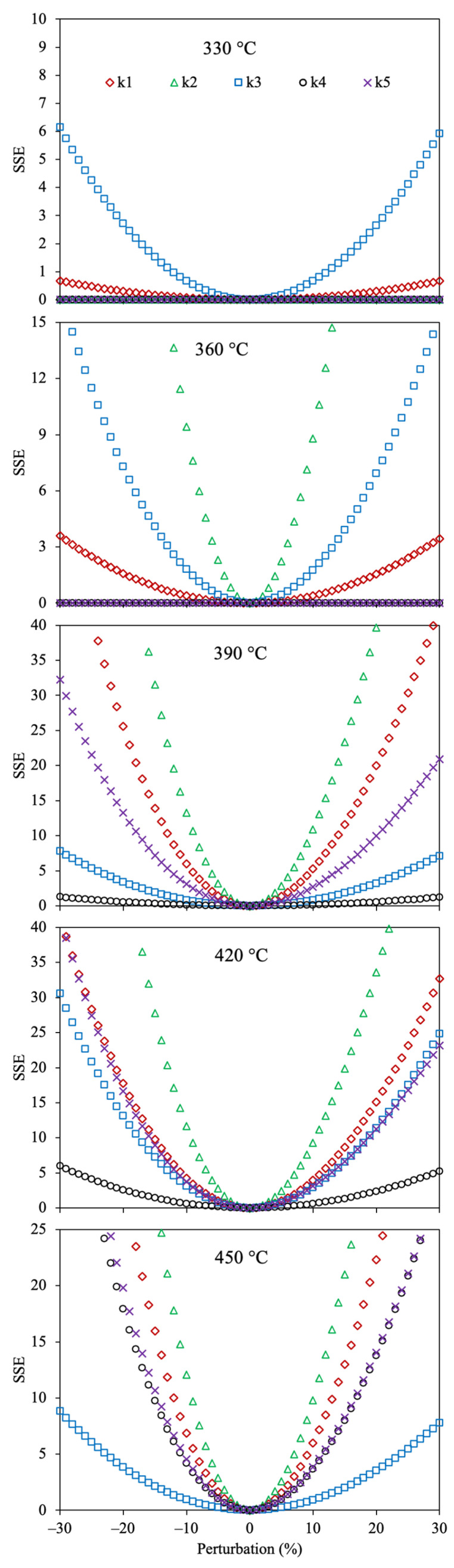
Sensitivity analysis for the estimated reaction rate coefficients using the 4-lump reaction scheme, Method 1, and SSE as the OF.

Figure 4 shows the sensitivity analysis for the determined kinetic parameters of the 4-lump network using Method 1 and SSE.

**Figure 5:**
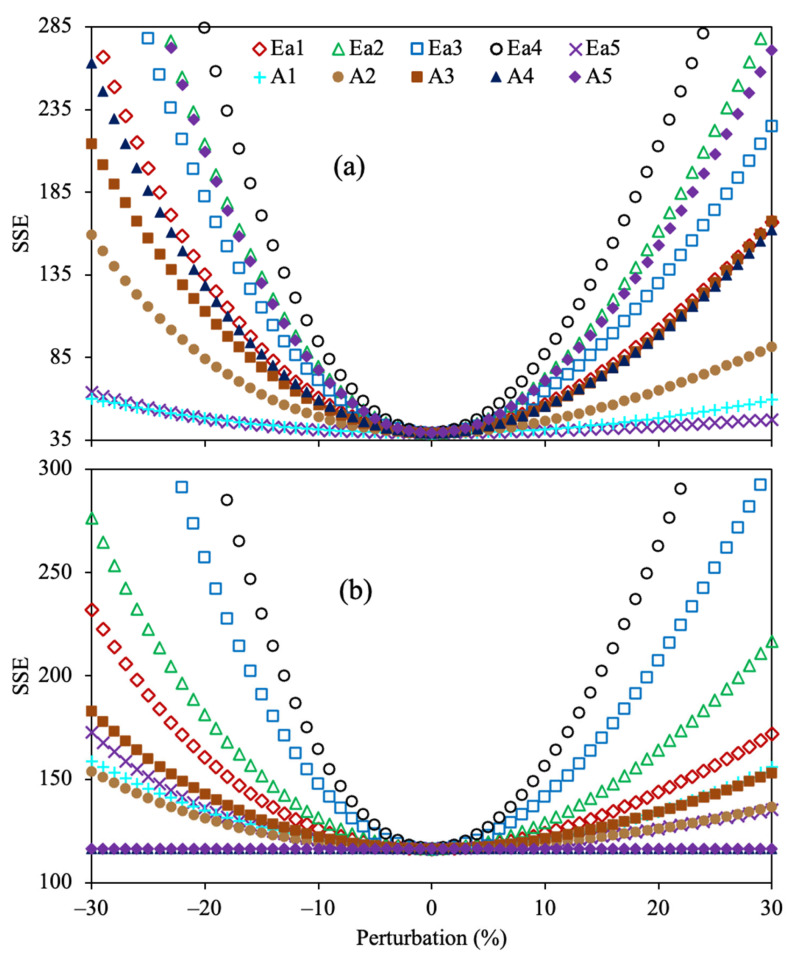
Sensitivity analysis for the estimated kinetic parameters using the 4-lump reaction scheme, SSE as the OF, and Method 2 (**a**) and Method 3 (**b**).

All the reaction rate coefficients obtained with this optimization approach displayed the lowest value of SSE at 0% perturbation, demonstrating that these values are optimal. It is observed that the kinetic parameter (*k*_4_) corresponding to the reaction of maltenes to gas does not affect the SSE at low temperatures (330–390 °C) since the combination of the low yield of gas and the small value of this parameter does not remarkably affect the OF. However, as the temperature increases (>420 °C), a small change in this reaction rate coefficient (*k*_4_) extraordinarily alters the accuracy owing to the increment in gas yield under these conditions. The sensitivity analysis for the optimized Arrhenius parameters of the 4-lump reaction scheme using Methods 2 and 3, together with SSE as OF, is depicted in Figure 5.

The results obtained agree that the estimated values for all parameters are optimal for both methods. Additionally, as well as in the previous results, the activation energy (*Ea*_4_) involving the gas formation from maltenes showed the highest sensitivity, suggesting that a minor alteration greatly modifies the fit.

### 2.2. Exhaustive Reaction Scheme

Based on the results after evaluating diverse optimization approaches, a more complex (6-lump) reaction scheme (Figure 1b) is studied using Method 1 and the SSE as the OF. The estimated reaction rate coefficients for the 6-lump reaction scheme using the optimization Method 1 and SSE as OF are reported in Table 3. Only the formation of coke is not performed (from both asphaltenes and polar aromatics) at 330 °C since the corresponding reaction rate coefficients are zero, which coincides with the lack of coke formation at 350 °C reported by Savage et al. [39]. It is observed that for asphaltenes conversion, larger values are obtained for the gas generation at low temperatures (330 and 360 °C), which is the first step in asphaltene pyrolysis, as aforementioned. Under the same conditions, the cracking of polar aromatics mainly produces naphthene aromatics based on the greater value of the related parameters. The reduction in maltene yield, accompanied by the increment in maltenes with lower molecular weight (light maltenes) at 350 and 450 °C, was described by Akmaz and Yasar [64] because the secondary pyrolysis reactions can justify this behavior. However, at temperatures over 390 °C, the coke formation from asphaltenes and polar aromatics thermal cracking predominates over other reactions. Coking, an extended pyrolysis transformation, fractionates large aromatic cores, such as asphaltenes and resins (or polar aromatics), into a carbon-rich core fraction and hydrogen-rich lower molecular weight compounds [61,65]. Usually, coke formation in the conversion of heavy feeds (heavy oil, tar, asphaltenes, etc.) occurs at temperatures over 400 °C due to the increase in the free radical production because the concentration of these highly reactive molecules is boosted at temperatures higher than 350 °C [66,67]. Saturate fraction is principally generated from naphthene aromatics at all temperatures according to the corresponding reaction rate coefficients. Similarly, the opposite reaction is preferred over the cracking of saturates to produce gases under all the conditions studied. Zhao et al. [40] reported that the cyclization of aliphatic side hydrocarbons is performed during asphaltene pyrolysis. Regarding the calculated activation energies, usually, the reactions for asphaltenes conversion exhibit larger values than other fractions, similar to those calculated for the 4-lump network using Method 1. Asphaltene molecules are more difficult to decompose as the conversion proceeds. The activation energies of maltene compounds (polar aromatics, naphthene aromatics, and saturates) displayed greater values for the polyaddition reactions rather than cracking. Trejo et al. [68] reported that the polyaddition reactions of maltene compounds, such as resins, through free radical production are enhanced at high temperatures (>400 °C), which corresponds to the higher activation energies for the recombination reactions for maltene fractions. A good accuracy of the 6-lump kinetic model is demonstrated by the low SSE values obtained at all temperatures.

**Figure 6:**
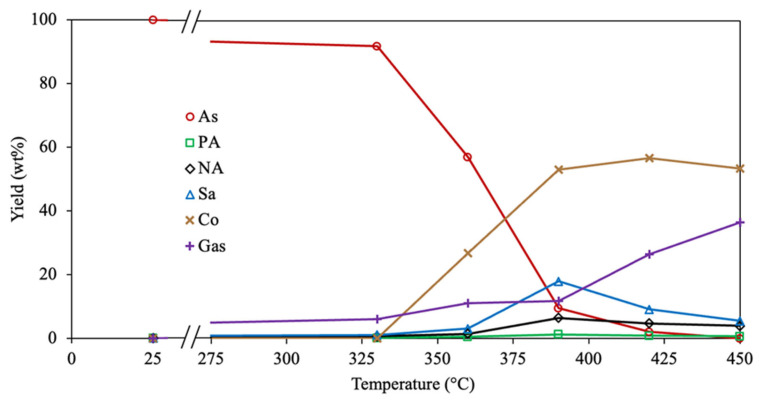
Experimental (symbols) and calculated (lines) yields for all compounds involved in the 6-lump reaction scheme employing Method 1 and SSE as the OF.

The predicted and experimental data using the estimated kinetic parameters are observed in Figure 6.

The experimental yields of polar aromatics, naphthene aromatics, and saturates exhibit a similar trend to the maltene fraction since their content increases as the temperature is under 390 °C. Subsequently, the yield of these hydrocarbons is diminished with a further temperature rise. This suggests that the same reactions (cracking, dealkylation, dehydrogenation, and recombination) are performed in the diverse maltene compounds during asphaltene pyrolysis. The 6-lump kinetic model accurately represents the reactions performed by all compounds because of the low difference between the experimental and calculated points, achieving an excellent fit.

**Figure 7:**
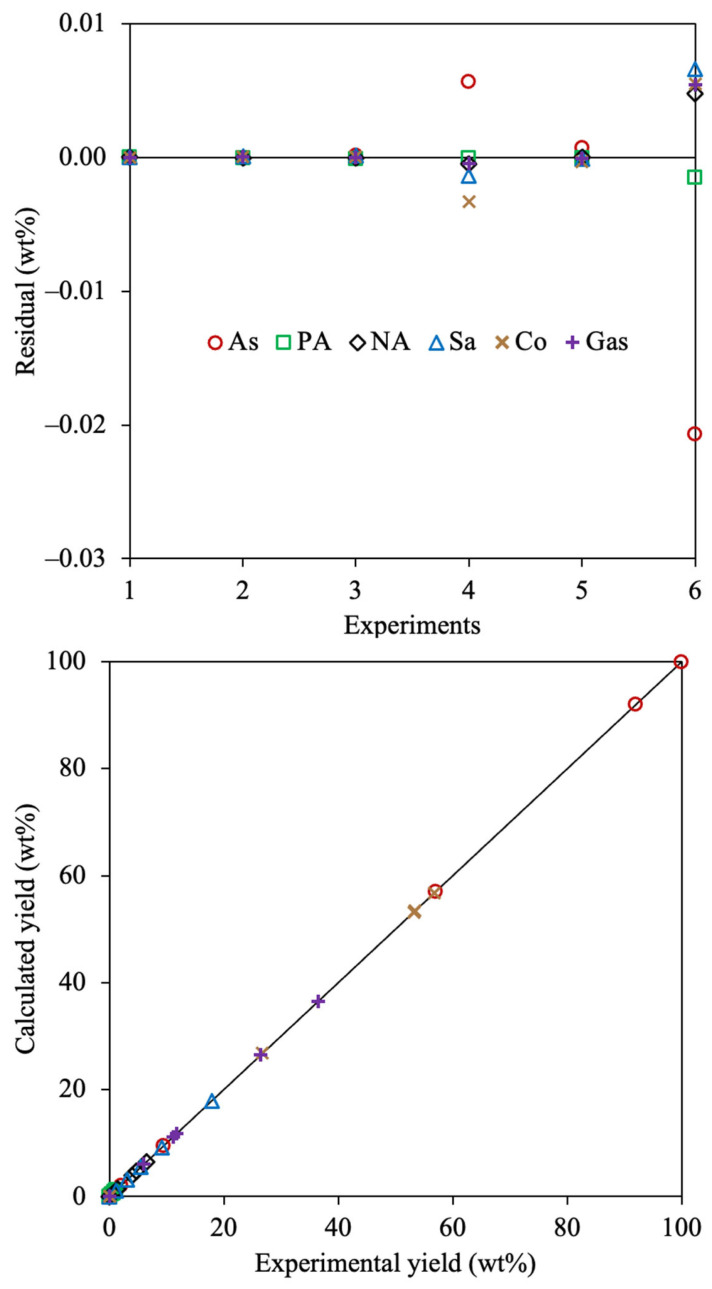
Residual and parity plots for the estimated 6-lump kinetic model using Method 1 and SSE as the OF.

The statistical analysis for the 6-lump reaction network is depicted in Figure 7.

The random and equal distribution of the residuals indicates that the parameter estimation process was appropriately carried out. Asphaltenes, coke, and saturates displayed the furthest residuals among all compounds, these being the fractions with higher yields, thus the larger difference between the calculated and analyzed values. It can be observed that the lower residuals are obtained using the 6-lump scheme than those estimated employing the 4-lump network. This indicates that a more exhaustive reaction scheme allows a better representation of all performed reactions that undergo all the involved fractions. The parity plot corroborates the outstanding agreement between the calculated and experimental yields using the estimated kinetic parameters for the exhaustive reaction scheme.

**Figure 8:**
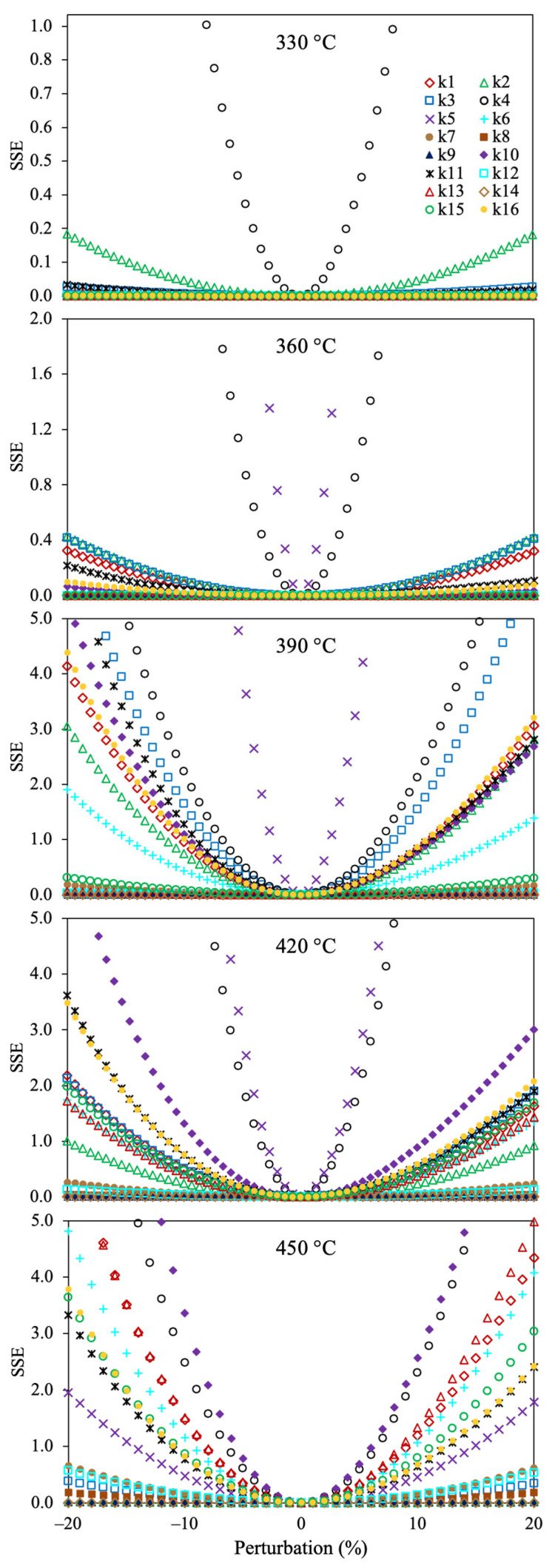
Sensitivity analysis for the estimated reaction rate coefficients using the 6-lump reaction scheme, Method 1, and SSE as the OF.

Figure 8 shows the sensitivity analysis for the optimized reaction rate coefficients of the 6-lump reaction scheme. It can be noticed that all the estimated kinetic parameters give the lowest value of the SSE at 0% of perturbation, demonstrating that these values are optimal. The reaction rate coefficients involving the asphaltene conversion into gases and coke (*k*_4_ and *k*_5_) are the most sensitive parameters (a small change increases the OF value). On the contrary, the kinetic parameters with lower susceptibility for raising the SSE include the production of asphaltenes from polar and naphthene aromatics. The reason may be owed to the large yield of asphaltenes, gas, and coke, and their dependency on the temperature with the thermal production of these pseudocomponents.

The primary distinction between this study and previously reported kinetic models lies in the implementation of a more intricate reaction scheme. This enhanced framework effectively captures the behavior of the liquid products generated during the pyrolysis of asphaltenes. A thorough comparison of the results from this research with existing kinetic models in the literature reveals a significant finding: not only does the asphaltene fraction undergo polyaddition reactions that ultimately lead to coke formation, but the maltenes fractions also exhibit a tendency to recombine as the temperature increases. This observation suggests a complex interplay during the coking process. Specifically, it indicates that coking primarily initiates through the recombination of asphaltenes below 390 °C. However, once this critical temperature threshold is exceeded, polyaddition reactions involving naphthenes and polar aromatics become increasingly prominent, resulting in heightened coke formation due to the augmented presence of condensed core free radicals.

The physical behavior of asphaltenes is intricately governed by a delicate balance of intermolecular forces among all the molecules present in the system. This balance is influenced not only by the structural properties of the molecules themselves but also by the number and nature of the elements involved, particularly the presence of metals and heteroatoms [47,57]. Asphaltenes can be classified into two primary types based on the average strata: archipelago and island asphaltenes. The former comprises a network of small aromatic polycyclic hydrocarbons linked by flexible aliphatic chains. This configuration creates a somewhat dispersed structure that affects their physical properties. On the other hand, island asphaltenes showcase a distinctive architecture characterized by a solitary, large aromatic core, surrounded by aliphatic chains that extend from the periphery [69,70,71]. The morphology of these asphaltenes significantly influences their behavior in different environments. Archipelago-type asphaltenes tend to display a more gradual aggregation process due to their more dispersed nature. In contrast, island-type asphaltenes, with their higher concentration of condensed polyaromatic hydrocarbons, tend to aggregate more rapidly. This quick aggregation is attributed to their compact structure, which encourages closer interactions among the molecules, as mentioned above [69,72].

## 3. Materials and Methods

### 3.1. Experimental Data

The data used to develop the kinetic models are obtained from experimental tests reported by Afanasjeva et al. [73]. The availability of this high-fidelity data—encompassing both general lumped fractions and detailed product distributions—is vital to develop a more comprehensive and mechanistically insightful reaction scheme. The asphaltenes utilized in this study were extracted from a heavy crude oil with a low API gravity of 9.6°, which contained a significant concentration (20.8 wt.%) of this heavy fraction. Comprehensive characterization revealed the complex nature of the isolated asphaltenes, which exhibited a high average molecular weight of 1492 g/mol. Further structural analysis, including NMR spectroscopy, provided key molecular parameters: an aromaticity factor of 0.4 and a hypothetical core structure comprising a polycyclic aromatic system of 10 fused aromatic carbons, surrounded by 26 peripheral aliphatic carbons. The isolated asphaltene pyrolysis experiments were conducted in a batch reactor at temperatures from 330 to 450 °C under an inert atmosphere (N_2_). The reaction was performed under non-isothermal conditions, elevating the temperature from a starting point of 25 °C to the targeted pyrolysis temperature at a controlled heating rate of 5 °C/min. As the temperature reached the desired value, the reactor was removed from the external electric heater and cooled down with cold water, quenching the reaction. Subsequently, the reactor content was meticulously collected and categorized into various fractions: unreacted asphaltenes, solid coke, liquid products, and non-condensable gases. The solid residue within the reactor was subjected to extraction using toluene in a Soxhlet apparatus, recovering the soluble fraction (asphaltenes) and the insoluble fraction (coke). Additionally, the composition of the liquid product was fractionated into saturates, naphthene, and polar aromatic hydrocarbons, commonly referred to as maltenes in the SARA analysis, according to the ASTM D 4124-09 standard method.

### 3.2. Kinetic Modeling

To evaluate the optimal approach for kinetic parameter estimation, a four-lump reaction scheme (Figure 1a) is used, contemplating the transformation between asphaltenes, maltenes, coke, and gas, as proposed by Yasar et al. [32].

The thermal cracking of asphaltenes produces maltenes and gas, while some of the originated free radicals undergo recombination reactions to generate coke. Furthermore, maltenes also suffer decomposition and polyaddition transformations to form gases and asphaltenes, respectively. The first-order reaction is usually reported for asphaltene pyrolysis [32,68,74,75], which is adopted in this work. According to this, the differential equation system is derived as follows:

*Asphaltenes (As):*

(1) $$\frac{dy_{As}}{dt}=-\left(k_{1}+k_{2}+k_{3}\right)y_{As}+k_{5}y_{Ma}$$

*Maltenes (Ma):*

(2) $$\frac{dy_{Ma}}{dt}=k_{1}y_{As}-\left(k_{4}+k_{5}\right)y_{Ma}$$

*Coke (Co):*

(3) $$\frac{dy_{Co}}{dt}=k_{2}y_{As}$$

*Gases:*

(4) $$\frac{dy_{Gas}}{dt}=k_{3}y_{As}+k_{4}y_{Ma}$$

where $y_{i}$ is the yield of component *i*, t is the reaction time, and $k_{j}$ is the reaction rate coefficient for reaction *j*. The optimization of the reaction rate coefficients in these Equations (1)–(4) is denoted as Method 1. The reaction system represents the transformation of the corresponding compounds with time. However, the low number of experiments varying the reaction time at the same temperature may decrease the accuracy of predicting the related yields. Therefore, the Arrhenius Equation (5) is substituted in each of the equations to calculate the change in composition with temperature. Subsequently, the following mass balance equations can be obtained:

(5) kj=AjeEajRT

(6) dyAsdT=−A1eEa1RT+A2eEa2RT+A3eEa3RTyAs+A5eEa5RTyMa

(7) dyMadT=A1eEa1RTyAs−A4eEa4RT+A5eEa5RTyMa

(8) dyCodT=A2eEa2RTyAs

(9) dyGasdT=A3eEa3RTyAs+A4eEa4RTyMa

where $A_{j}$ is the collision factor for reaction *j*, ${Ea}_{j}$ is the activation energy for reaction *j*, *R* is the universal gas constant, and *T* is the temperature. Method 2 is based on optimizing the Arrhenius parameters (collision factors and activation energies) in Equations (6)–(9).

According to Rhim et al. [76], the time and temperature can be related if the heating rate increases constantly, as in the experiments for asphaltene pyrolysis conducted by Afanasjeva et al. [73]. Therefore, a linear correlation (Equation (10)) can be employed to obtain the following mass balance equations:

(10) $$T=T_{0}+at$$

(11) dyAsdt=−A1eEa1RT0+at+A2eEa2RT0+at+A3eEa3RT0+atyAs+A5eEa5RT0+atyMa

(12) dyMadt=A1eEa1RT0+atyAs−A4eEa4RT0+at+A5eEa5RT0+atyMa

(13) dyCodt=A2eEa2RT0+atyAs

(14) dyGasdt=A3eEa3RT0+atyAs+A4eEa4RT0+atyMa

where a is the heating rate, and $T_{0}$ is the initial temperature. The optimization methodology for the Arrhenius parameters based on these equations is considered in Method 3.

The kinetic study on the chemical transformation of the involved hydrocarbons during asphaltene pyrolysis is performed using a 6-lump reaction scheme (Figure 1b). This is done by separating the maltene fraction into saturates (Sa), naphthene aromatics (NA), and polar aromatics (PA). This reaction network includes the same cracking and polyaddition reactions of asphaltenes but with larger liquid products (polar aromatics, naphthene aromatics, and saturates) instead of only maltenes. Gas production is carried out by decomposing all SARA (asphaltenes, polar aromatics, naphthene aromatics, and saturates) fractions. The coke formation is due to the recombination of free radicals produced from asphaltene and polar aromatic cracking. Furthermore, this scheme considers the in-series disintegration reactions of SARA fractions from heavier toward lighter hydrocarbons and the backward transformations (polyaddition). Additionally, the parallel cracking of asphaltenes to produce naphthene aromatics and saturates is contemplated, as well as the recombination of naphthene aromatics into asphaltenes. The corresponding reaction rate equations can be derived as follows:

(15) $$\frac{dy_{As}}{dt}=-\left(k_{1}+k_{2}+k_{3}+k_{4}+k_{5}\right)y_{As}+k_{9}y_{PA}+k_{14}y_{NA}$$

(16) $$\frac{dy_{PA}}{dt}=k_{1}y_{As}-\left(k_{6}+k_{7}+k_{8}+k_{9}+k_{10}\right)y_{PA}+k_{13}y_{NA}$$

(17) $$\frac{dy_{NA}}{dt}=k_{2}y_{As}+k_{6}y_{PA}-\left(k_{11}+k_{12}+k_{13}+k_{14}\right)y_{NA}+k_{16}y_{Sa}$$

(18) $$\frac{dy_{Sa}}{dt}=k_{3}y_{As}+k_{7}y_{PA}+k_{11}y_{NA}-\left(k_{15}+k_{16}\right)y_{Sa}$$

(19) $$\frac{dy_{Gas}}{dt}=k_{4}y_{As}+k_{8}y_{PA}+k_{12}y_{NA}+k_{15}y_{Sa}$$

(20) $$\frac{dy_{Co}}{dt}=k_{5}y_{As}+k_{10}y_{PA}$$

### 3.3. Parameter Estimation

**Figure 9:**
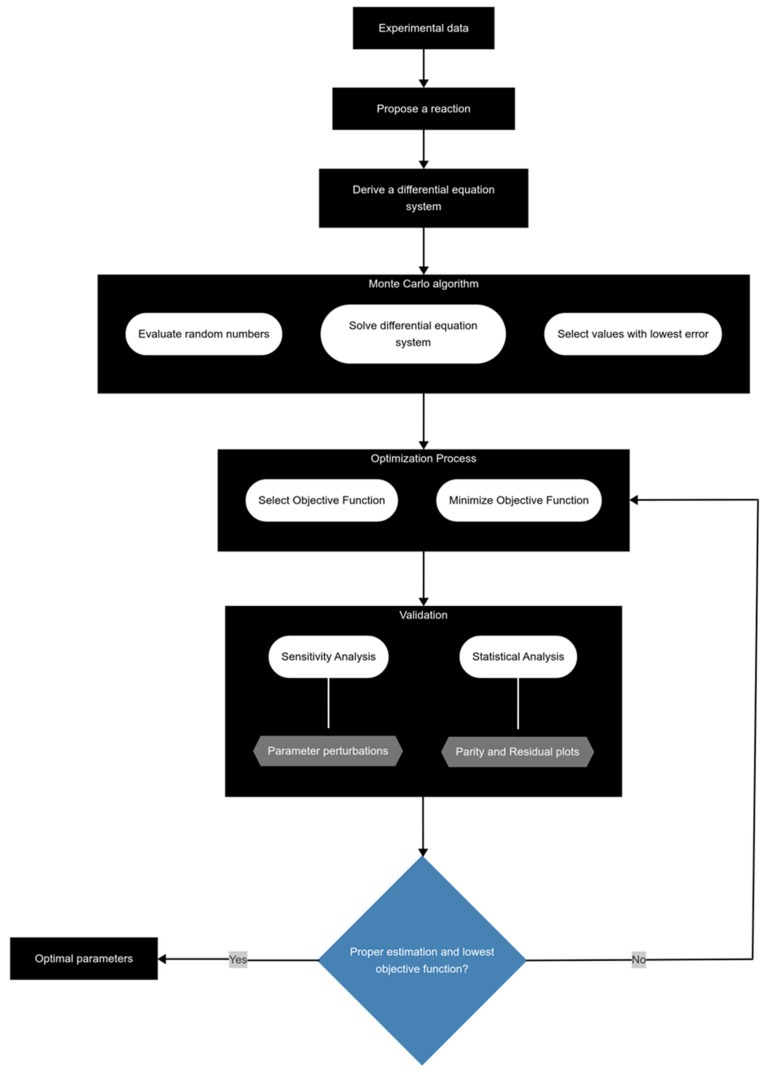
Methodology for proper parameter estimation during kinetic modeling.

The parameters were estimated through a series of steps (Figure 9) to corroborate that the obtained values are optimal using MATLAB (R2023a).

The process begins with the acquisition of experimental data and the proposal of a reaction scheme. After this, kinetic parameter values are estimated through the Monte Carlo algorithm, which utilizes random numbers to generate initial guesses. These guesses are then evaluated by solving a system of differential equations with the *ode45* function to calculate the objective function (OF) and select those values with lower error. Different equations have been reported as OF, such as the average absolute error (AAE) and the sum of square error (SSE), with the latter being the most used and adopted in this work.

(21) $$AAE(\%)=\frac{\sum_{j=1}^{nr}\sum_{i=1}^{nc}\left(\frac{\left|y_{i,j}^{exp}-y_{i,j}^{cal}\right|}{y_{i,j}^{exp}}\right)x100}{N}$$

(22) $$SSE=\sum_{j=1}^{nr}\sum_{i=1}^{nc}{\left(y_{i,j}^{cal}-y_{i,j}^{exp}\right)}^{2}$$

(23) $$k_{j}\geq 0$$

(24) $$k_{j}\;at\;T_{2}>k_{j}\;at\;T_{1}\;{for\;T}_{2}>T_{1}$$

where $y_{i,j}^{cal}$ and $y_{i,j}^{exp}$ are the calculated and experimental yields of component *i* in reaction *j*, respectively, *nr* and *nc* are the number of reactions and pseudocomponents, respectively, ${\bar{y}}_{i,j}^{exp}$ is the average experimental yields of component *i* in reaction *j*, and *N* is the number of observations. Following this, the selected parameters are employed in the optimization process, which mirrors the same steps but focuses on minimizing the OF using the *lsqnonlin* function, which implements a trust-region reflective algorithm. This optimization is adjusted to specific constraints. Finally, the estimated kinetic parameters undergo sensitivity and statistical analyses. The methodology involves systematically perturbing the parameter values, re-evaluating the objective function (OF), and generating plots to assess the sensitivity and stability of the optimization outcomes. To further validate the reliability of the results, statistical analyses, including parity and residual plots, are conducted, ensuring both the effectiveness of the optimization procedure and the robustness of the fitted model.

The precision of the estimated reaction rate coefficients (*k_i_*) was quantified by calculating their respective 95% confidence intervals. This statistical procedure was employed to assess the uncertainty associated with each parameter estimate derived from the kinetic model of the experimental data. The confidence intervals were constructed according to established statistical protocols for nonlinear regression, as reported elsewhere [77,78]. The methodology leverages the standard error (*se_i_*) of each estimated coefficient, which serves as a measure of its estimated standard deviation. This standard error is then scaled by a critical value derived from the inverse of Student’s t distribution. The specific critical value, denoted as t_(α/2, n–p)_, is determined by the chosen confidence level (1 − α) of 95% (thus, α = 0.05) and the degrees of freedom available for the regression, which is calculated as the difference between the number of experimental observations (*n*) and the number of fitted parameters (*p*). The resulting bilateral confidence interval for any given reaction rate coefficient, *k_i_*, is therefore given by:

(25) ki ± seit(α2,n−p)

## 4. Conclusions

The evaluation of diverse parameter estimation approaches, objective functions, and reaction schemes is studied during the kinetic modeling of asphaltenes pyrolysis. Three optimization methodologies are assessed based on reaction rate coefficients and Arrhenius parameters, together with two different objective functions (SSE and AAE). The proposed reaction schemes comprise the pathways between asphaltenes, maltenes, gases, and coke in 4-lump and 6-lump models, contemplating the separation of maltenes into polar aromatics, naphthene aromatics, and saturates. Based on the results, both assessed networks (4-lump and 6-lump) exhibit adequate accuracy; however, a more exhaustive reaction scheme increases the precision of predicting all experimental yields. Using SSE as OF and optimizing the reaction rate coefficients at each temperature (estimation Method 1) exhibits better accuracy during the kinetic modeling of asphaltene pyrolysis. In addition, employing AAE as OF heightens the uncertainty associated with the estimated kinetic parameters, which is mitigated by incorporating SSE in the optimization process, achieving appropriate confidence intervals.

The statistical and sensitivity analyses corroborated that all the estimated kinetic parameters are adequately optimized and exhibit optimal values. According to the obtained kinetic parameters, temperature affects the selectivity of asphaltene pyrolysis: at low temperatures (330–390 °C), the production is toward maltenes and gases, while at elevated temperatures (>390 °C), coke formation is favored. Prolonging time and high-temperature exposure cause secondary cracking to the generated maltenes. The cleavage of side aliphatic chains attached to aromatic rings, the aromatization of saturated aromatics, the polyaddition of free radicals, and the peri-condensation of highly compressed cores predominate under pyrolysis conditions. Although this study accurately represents all these reactions that asphaltenes undergo, it is restricted to modeling the pyrolysis behavior of isolated asphaltenes due to their reactivity and aggregation characteristics, which are influenced by environmental factors and morphological properties.

## Figures and Tables

**Table 1:** Estimated reaction rate coefficients (min^−1^) for the 4-lump kinetic model using AAE as the objective function.

Parameter			Temperature (°C)			Ea (kcal/mol)	A (1/min)
	**330**	**360**	**390**	**420**	**450**		
			Method 1				
*k*_1_	3.4112 × 10^−4^ ± 1.22 × 10^−5^	1.0004 × 10^−3^ ± 5.34 × 10^−4^	1.8978 × 10^−1^ ± 1.7081	2.7366 × 10^−1^ ± 1.7158	4.7483 × 10^−1^ ± 2.77 × 10^−3^	58.69	6.4007 × 10^17^
*k*_2_	1.7083 × 10^−9^ ± 2.66 × 10^−9^	5.2253 × 10^−3^ ± 1.44 × 10^−4^	4.2844 × 10^−1^ ± 2.2499	9.7593 × 10^−1^ ± 2.4467	1.8766 ± 2.77 × 10^−3^	139.45	8.2484 × 10^43^
*k*_3_	1.0257 × 10^−3^ ± 3.34 × 10^−5^	2.1566 × 10^−3^ ± 1.11 × 10^−3^	6.5875 × 10^−2^ ± 1.8688	4.0989 × 10^−1^ ± 2.1882	1.1606 ± 2.77 × 10^−3^	55.80	1.0823 × 10^17^
*k*_4_	2.0747 × 10^−4^ ± 5.93 × 10^−5^	5.5793 × 10^−4^ ± 4.19 × 10^−3^	1.1112 × 10^−3^ ± 5.69 × 10^−1^	1.4700 × 10^−3^ ± 5.89 × 10^−1^	3.3770 × 10^−3^ ± 3.75 × 10^−4^	18.99	1.7564 × 10^3^
*k*_5_	1.5921 × 10^−7^ ± 5.93 × 10^−5^	4.7623 × 10^−7^ ± 7.29 × 10^−3^	9.5244 × 10^−7^ ± 4.92 × 10^−1^	1.8098 × 10^−6^ ± 3.74 × 10^−1^	3.5834 × 10^−6^ ± 5.19 × 10^−6^	21.93	1.5552 × 10^1^
AAE (%)	4.25 × 10^−2^	4.96 × 10^−4^	29.45	25.89	4.88 × 10^−2^		
			Method 2				
*k*_1_	3.1054 × 10^−4^	2.0277 × 10^−3^	1.1173 × 10^−2^	5.3110 × 10^−2^	2.2183 × 10^−1^	47.46 ± 9.47 × 10^5^	4.9023 × 10^13^ ± 8.79 × 10^5^
*k*_2_	1.6630 × 10^−9^	5.5867 × 10^−7^	1.1088 × 10^−4^	1.3921 × 10^−2^	1.1704	147.15 ± 9.45 × 10^5^	3.4505 × 10^44^ ± 9.49 × 10^5^
*k*_3_	1.7331 × 10^−4^	1.9270 × 10^−3^	1.7230 × 10^−2^	1.2745 × 10^−1^	7.9855 × 10^−1^	60.93 ± 8.52 × 10^5^	2.0691 × 10^18^ ± 9.49 × 10^5^
*k*_4_	5.2900 × 10^−69^	1.3030 × 10^−65^	1.5835 × 10^−62^	1.0405 × 10^−59^	3.9912 × 10^−57^	197.54 ± 6.03 × 10^5^	2.0005 × 10^3^ ± 9.47 × 10^5^
*k*_5_	6.3042 × 10^−8^	1.6988 × 10^−7^	4.1849 × 10^−7^	9.5356 × 10^−7^	2.0292 × 10^−6^	25.08 ± 9.28 × 10^5^	7.6812 × 10^1^ ± 9.45 × 10^5^
AAE (%)	23.52	68.42	128.75	105.14	6.77 × 10^−2^		
			Method 3				
*k*_1_	3.7627 × 10^−4^	3.8251 × 10^−3^	3.1526 × 10^−2^	2.1647 × 10^−1^	1.2668	58.66 ± 1.16 × 10^7^	6.7805 × 10^17^ ± 1.25 × 10^7^
*k*_2_	3.8453 × 10^−7^	9.5316 × 10^−5^	1.4348 × 10^−2^	1.3993	9.3321 × 10^1^	139.45 ± 1.24 × 10^7^	1.3042 × 10^44^ ± 1.15 × 10^7^
*k*_3_	6.4025 × 10^−4^	5.8169 × 10^−3^	4.3284 × 10^−2^	2.7071 × 10^−1^	1.4542	55.82 ± 1.20 × 10^7^	1.0769 × 10^17^ ± 1.13 × 10^7^
*k*_4_	1.8256 × 10^−2^	3.1931 × 10^−2^	5.3095 × 10^−2^	8.4486 × 10^−2^	1.2935 × 10^−1^	14.14 ± 9.83 × 10^6^	2.4321 × 10^3^ ± 1.25 × 10^7^
*k*_5_	1.7225 × 10^−7^	4.0969 × 10^−7^	9.0092 × 10^−7^	1.8505 × 10^−6^	3.5807 × 10^−6^	21.92 ± 1.25 × 10^7^	1.5052 × 10^1^ ± 1.23 × 10^7^
AAE (%)	123.02	84.46	227.15	45.07	37.03		

**Table 2:** Estimated reaction rate coefficients (min^−1^) for the 4-lump kinetic model using SSE as the objective function.

Parameter			Temperature (°C)			Ea (kcal/mol)	A (1/min)
	**330**	**360**	**390**	**420**	**450**		
			Method 1				
*k*_1_	3.3903 × 10^−4^ ± 2.20 × 10^−7^	1.0004 × 10^−3^ ± 1.01 × 10^−5^	3.1691 × 10^−2^ ± 5.01 × 10^−4^	1.1432 × 10^−1^ ± 8.61 × 10^−4^	2.2679 ± 0.7202	64.26	3.5839 × 10^19^
*k*_2_	4.6196 × 10^−9^ ± 1.36 × 10^−9^	5.2253 × 10^−3^ ± 4.94 × 10^−7^	2.5741 × 10^−2^ ± 1.45 × 10^−4^	7.9198 × 10^−2^ ± 3.29 × 10^−4^	9.2485 × 10^−1^ ± 0.4162	121.27	2.9042 × 10^37^
*k*_3_	1.0263 × 10^−3^ ± 1.55 × 10^−7^	2.1566 × 10^−3^ ± 7.10 × 10^−6^	4.1427 × 10^−3^ ± 3.63 × 10^−4^	2.5740 × 10^−2^ ± 7.91 × 10^−4^	1.9433 × 10^−1^ ± 0.6680	36.92	1.4069 × 10^10^
*k*_4_	1.0059 × 10^−5^ ± 1.47 × 10^−5^	5.5793 × 10^−4^ ± 1.95 × 10^−4^	1.6102 × 10^−3^ ± 3.58 × 10^−4^	3.7999 × 10^−3^ ± 2.27 × 10^−4^	9.9379 × 10^−3^ ± 0.0117	46.16	1.5204 × 10^12^
*k*_5_	1.6063 × 10^−7^ ± 1.48 × 10^−6^	4.7623 × 10^−7^ ± 1.95 × 10^−4^	1.8464 × 10^−2^ ± 3.17 × 10^−4^	2.8000 × 10^−2^ ± 2.01 × 10^−4^	3.7722 × 10^−2^ ± 4.1 × 10^−5^	104.48	1.0204 × 10^31^
SSE	4.20 × 10^−9^	8.92 × 10^−8^	3.41 × 10^−5^	1.37 × 10^−7^	1.71 × 10^−2^		
			Method 2				
*k*_1_	6.4818 × 10^−6^	1.3261 × 10^−3^	1.6763 × 10^−1^	1.3938	8.0310 × 10^2^	134.60 ± 16.11	3.8246 × 10^43^ ± 13.33
*k*_2_	5.0881 × 10^−4^	5.1424 × 10^−3^	4.2158 × 10^−2^	2.8807 × 10^−1^	1.6783	58.51 ± 12.37	8.1056 × 10^17^ ± 7.47
*k*_3_	1.0456 × 10^−3^	1.8830 × 10^−3^	3.2154 × 10^−3^	5.2420 × 10^−3^	8.2064 × 10^−3^	14.88 ± 10.37	2.5803 × 10^2^ ± 5.32
*k*_4_	8.9630 × 10^−4^	1.8651 × 10^−3^	3.6320 × 10^−3^	6.6763 × 10^−3^	1.1668 × 10^−2^	18.54 ± 8.18	4.6660 × 10^3^ ± 18.11
*k*_5_	1.8023 × 10^−4^	3.8287 × 10^−3^	6.1687 × 10^−2^	7.8134 × 10^−1^	8.0168	77.31 ± 16.47	1.8487 × 10^24^ ± 13.84
SSE	13.76	2.16	10.58	10.52	2.32		
			Method 3				
*k*_1_	3.5754 × 10^−3^	4.2966 × 10^−2^	4.1231 × 10^−1^	3.2532	2.1626 × 10^1^	62.89 ± 30.33	2.2002 × 10^20^ ± 28.87
*k*_2_	1.7249 × 10^−2^	8.3937 × 10^−2^	3.5397 × 10^−1^	1.3179	4.3995	40.03 ± 23.83	5.4923 × 10^12^ ± 38.03
*k*_3_	5.5058 × 10^−3^	9.1141 × 10^−3^	1.4415 × 10^−2^	2.1911 × 10^−2^	3.2168 × 10^−2^	12.75 ± 11.82	2.2938 × 10^2^ ± 27.74
*k*_4_	1.7000 × 10^−2^	3.2677 × 10^−2^	5.9206 × 10^−2^	1.0189 × 10^−1^	1.6763 × 10^−1^	16.53 ± 9.52	1.6598 × 10^4^ ± 2.28 × 10^3^
*k*_5_	1.4490 × 10^−13^	1.5655 × 10^−12^	1.3637 × 10^−11^	9.8492 × 10^−11^	6.0374 × 10^−10^	60.20 ± 32.91	9.4362 × 10^8^ ± 2.28 × 10^3^
SSE	43.46	23.23	23.83	14.65	11.15		

**Table 3:** Estimated reaction rate coefficients (min^−1^) for the 6-lump kinetic model using SSE as the objective function.

Parameter			Temperature (°C)			Ea (kcal/mol)	A (1/min)
	**330**	**360**	**390**	**420**	**450**		
*k*_1_	3.8296 × 10^−5^	4.2787 × 10^−4^	7.4244 × 10^−3^	1.0826 × 10^−2^	2.0579 × 10^−1^	59.07	1.0797 × 10^17^
*k*_2_	2.2709 × 10^−4^	4.2123 × 10^−4^	2.8826 × 10^−3^	3.6453 × 10^−3^	1.4074 × 10^−2^	30.06	1.5027 × 10^7^
*k*_3_	7.9086 × 10^−5^	3.9068 × 10^−4^	4.1168 × 10^−3^	4.9373 × 10^−3^	5.6074 × 10^−2^	45.26	1.9335 × 10^12^
*k*_4_	1.0212 × 10^−3^	2.1450 × 10^−3^	3.3553 × 10^−3^	1.0267 × 10^−2^	7.1234 × 10^−2^	28.61	1.6386 × 10^7^
*k*_5_	-	5.0019 × 10^−3^	1.4646 × 10^−2^	2.0360 × 10^−2^	4.3937 × 10^−2^	20.83	8.5888 × 10^4^
*k*_6_	8.7414 × 10^−3^	1.6010 × 10^−2^	3.0034 × 10^−2^	3.8788 × 10^−2^	8.6293 × 10^−2^	15.76	4.4239 × 10^3^
*k*_7_	2.1670 × 10^−3^	4.7991 × 10^−3^	9.4150 × 10^−3^	1.4715 × 10^−2^	3.0506 × 10^−2^	18.54	1.1564 × 10^4^
*k*_8_	4.5511 × 10^−4^	8.1044 × 10^−4^	1.6032 × 10^−3^	3.1944 × 10^−3^	6.3134 × 10^−3^	19.10	3.4302 × 10^3^
*k*_9_	1.4851 × 10^−4^	3.7298 × 10^−4^	7.4492 × 10^−4^	1.4927 × 10^−3^	2.9450 × 10^−3^	21.30	7.9311 × 10^3^
*k*_10_	-	3.8407 × 10^−2^	5.8946 × 10^−2^	8.9544 × 10^−2^	1.3103 × 10^−1^	12.43	7.4525 × 10^2^
*k*_11_	3.3203 × 10^−2^	5.8302 × 10^−2^	1.2149 × 10^−1^	2.0849 × 10^−1^	3.6737 × 10^−1^	17.57	7.3082 × 10^4^
*k*_12_	5.1758 × 10^−4^	7.3796 × 10^−4^	1.4569 × 10^−3^	3.2757 × 10^−3^	6.2550 × 10^−3^	18.58	2.2863 × 10^3^
*k*_13_	1.0549 × 10^−3^	1.6628 × 10^−3^	3.3985 × 10^−3^	1.9162 × 10^−2^	4.0538 × 10^−2^	27.88	9.1141 × 10^6^
*k*_14_	4.8665 × 10^−5^	1.7741 × 10^−4^	3.5495 × 10^−4^	7.3372 × 10^−4^	1.4308 × 10^−3^	23.76	2.3174 × 10^4^
*k*_15_	4.7533 × 10^−4^	8.9982 × 10^−4^	1.7477 × 10^−3^	6.5295 × 10^−3^	1.1447 × 10^−2^	24.00	1.9550 × 10^5^
*k*_16_	7.1213 × 10^−3^	2.1830 × 10^−2^	4.2320 × 10^−2^	1.1101 × 10^−1^	2.7702 × 10^−1^	25.83	1.6146 × 10^7^
SSE	2.22 × 10^−8^	5.59 × 10^−8^	4.56 × 10^−5^	6.16 × 10^−7^	5.56 × 10^−4^		

## Data Availability

Data will be available on request.
